# Effects of renin–angiotensin inhibitors on renal function and the clinical course in patients with decompensated cirrhosis

**DOI:** 10.1038/s41598-023-44299-w

**Published:** 2023-10-14

**Authors:** Tammo L. Tergast, Marie Griemsmann, Heiner Wedemeyer, Markus Cornberg, Benjamin Maasoumy

**Affiliations:** 1 Department of Gastroenterology, Hepatology, Infectious Diseases and Endocrinology, Hannover Medical School, Carl-Neuberg-Str. 1, 30625 Hannover, Germany; 2 German Centre for Infection Research, HepNet Study-House of the German Liver Foundation, 30625 Hannover, Germany; 3 Centre for Individualised Infection Medicine (CiiM), 30625 Hannover, Germany

**Keywords:** Liver cirrhosis, Hepatology

## Abstract

Patients with decompensated cirrhosis are at risk of developing acute kidney injury (AKI). Studies have suggested that inhibition of the Renin-Angiotensin System (RAS) has certain nephro- and hepatoprotective effects in patients with compensated liver disease. This study aimed to investigate the clinical impact of RAS-Inhibitors in individuals with decompensated liver cirrhosis. Overall, 1181 consecutive hospitalized patients with ascites that underwent paracentesis were considered for this retrospective study. In total, 667 patients with decompensated cirrhosis fulfilled the inclusion criteria and were finally analyzed. RAS-Inhibitor intake was documented in 41 patients (7%). First, 28-day incidences of AKI and grade III AKI of all patients with RAS-Inhibitors were compared to those without intake. Afterwards, propensity score matching was conducted in a 3:1 manner. Here, incidence of further renal endpoints such as need of hemodialysis were analyzed in detail. In the unmatched setting, intake of RAS-Inhibitors was not associated with an increased 28 day-incidence of AKI (P = 0.76) or LTx-free survival (P = 0.60). However, 28 day-incidence of grade III AKI was significantly lower in patients with RAS-Inhibitor intake (P < 0.001). In the matched setting, 28 day-incidence of AKI did not differ (P = 0.81), while grade III AKI was significantly less frequent in the RAS-Inhibitor group (P < 0.001). Need for hemodialysis was also significantly lower in patients with RAS-Inhibitors (P = 0.03) and LTx-free survival was comparable between both groups (P = 0.52). Thus, this study suggests that intake of RAS-Inhibitors is associated with decreased incidences of grade III AKI and need of hemodialysis in patients with decompensated liver disease.

## Introduction

Patients with decompensated liver disease are at a high risk of developing other non-liver organ failure^1^. Especially kidney function is often deteriorating in the context of hepatic decompensation and acute kidney injury (AKI) is a frequent complication associated with significantly decreased survival^2^. Furthermore, it has been shown how AKI can lead to a permanently decreased estimated glomerular filtration rate (eGFR) after AKI via induction of irreversible tubular necrosis^3^. Thus, studies focused on the identification of risk factors for hepatic decompensation and AKI. In this context, comorbidities such as type 2 diabetes mellitus or certain comedication like analgesics have already been identified as risk factors for AKI in patients with decompensated liver disease^4,5^.

Inhibitors of the Renin–Angiotensin–Aldosterone system (RAS-Inhibitors), especially Angiotensin II receptor antagonists and Angiotensin-converting enzyme (ACE) inhibitors, are popular in the non-cirrhotic normal population for the treatment of hypertension or heart failure with reduced ejection fraction^6^. Some studies have also suggested that RAS-Inhibitors exert nephroprotective effects and are associated with increased survival, even in patients on hemodialysis^7^. Additionally, antifibrotic characteristics of RAS-Inhibitors have been described in those with non-alcoholic fatty liver disease and one study found improved survival in CHILD A patients with hepatocellular carcinoma (HCC) and RAS-Inhibitor intake^8,9^. Therefore, RAS-Inhibitors may be beneficial in patients with earlier stages of liver disease and compensated liver disease, i.e. those with CHILD A cirrhosis. However, no studies have investigated the long-term impact of RAS-Inhibitors regarding clinical endpoints such as renal failure or survival in the setting of decompensated liver cirrhosis. Evidence in patients with decompensated cirrhosis is mostly limited to only one study including 6 patients with cirrhosis and ascites, in whom Captopril, an ACE-Inhibitor, was newly administered^10^. Based on this, current guidelines do not encourage the use of RAS-Inhibitors in patients with ascites^11^. Besides the potential induction of hypotension, RAS-Inhibitors result in a decrease of Angiotensin II. Angiotensin II is important for maintaining the intraglomerular hydrostatic pressure in settings of decreased renal blood flow^12^. In decompensated cirrhosis, renal blood flow can be impaired through micro- and macrocirculatory effects of cirrhosis related complications like ascites or inflammation^10,13^. Hence, intake of RAS-Inhibitors could further increase the risk of AKI in those with decompensated liver disease. If this is also the case in those with ongoing RAS-Inhibitor intake is currently unknown. Therefore, this study focused on the clinical effects of RAS-Inhibitor intake in patients with decompensated liver disease.

## Methods

### Study cohort

For this retrospective study, all hospitalized patients that underwent paracentesis between 2012 and 2018 at Hannover Medical School were considered. In- and exclusion criteria have been described previously^14^. Briefly, all patients without liver cirrhosis or with HIV, congenital immunodeficiency, history of non-liver organ transplantation and malignancies except for HCC within the MILAN criteria were excluded (Supplementary Fig. 1). Baseline was defined as time of first paracentesis, which was of diagnostic nature in every patient to rule out presence of spontaneous bacterial peritonitis (SBP).

### Data assessment

Laboratory values of included patients were automatically extracted from the clinical information system and carefully validated manually. Information regarding demographics, clinical endpoints and comedication such as intake of ACE-Inhibitors or Angiotensin II Antagonists was taken from the patients’ medical records. The diagnosis of AKI and severe AKI, i.e. AKI grade III, was made according to current EASL and IAC guideline 2, 11. AKI was defined as and Increase in sCr ≥ 26.5 µmol/l within 48 h or, A percentage increase sCr ≥ 50% which is known, or presumed, to have occurred within the prior seven days. Severe AKI was defined as increase of sCr > 3 fold from baseline or sCr ≥ 4.0 mg/dl (353.6 µmol/l) with an acute increase ≥ 0.3 mg/dl (≥ 26.5 µmol/l) or initiation of renal replacement therapy (RRT). Estimated glomerular filtration rate was calculated using the CKD-EPI formula.

### Study design

Primary endpoints of this study were short term incidences of AKI and severe AKI in the context of hepatic decompensation and long term LTx-free survival. Short term follow-up was defined as 28-day follow-up and long-term follow-up was defined as 365-day and/or 5-year follow-up. Therefore, AKI and severe AKI were analyzed within 28-days after first paracentesis, while LTx-free survival was analyzed in the context of 28-days, 365-days and 5-years of follow-up. In the matched setting, need for hemodialysis, change of eGFR within 365 days and long-term HCC incidence were added to the analyzed endpoints.

#### Analysis 1

After application of in- and exclusion criteria, all patients were analyzed in an unmatched approach. For this analysis, multivariable competing risk analysis was conducted to adjust for potential confounding factors. Included factors were age, leukocyte count, platelet count, presence of type II Diabetes Mellitus, mean arterial pressure and MELD score. In an additional analysis we adjusted for eGFR and INR instead for MELD score. All Included parameters were common risk factor for an unfavorable outcome or associated with disease severity in patients with liver cirrhosis^1,5^. Analyzed endpoints were LTx-free survival and incidence of AKI and severe AKI.

#### Analysis 2

Second, we matched patients with RAS-Inhibitor intake with patients without RAS-Inhibitor intake via propensity score matching (PPSM) in a 3:1 manner. Included factors in the matching process were age, leukocyte count, platelet count, presence of type II Diabetes Mellitus, mean arterial pressure and eGFR. Analyzed endpoints were short-term incidence of AKI, severe AKI and LTx-free survival, need for hemodialysis and HCC incidence in the follow-up. In this matched setting, univariate competing risk analysis was conducted.

### Statistics

Analyses were performed using R Version 4.2.1 (packages: ‘cmprsk,’ ‘RItools, MatchIt, Rcmdr), SPSS (IBM, Version 26) and GraphPad Prism (Version 7; GraphPad Software Inc.). Regarding unmatched data, continuous variables were analyzed using unmatched t-testing, while categorial variables were analyzed using Fisher’s exact or Chi-squared test. In matched settings, Cochrane-Mantel–Haenszel statistics was used for categorical data and repeated measures ANOVA for continuous variables. PPSM was conducted in a 3:1 manner via nearest neighbor matching with a Caliper of < 0.15. Kaplan–Meier curves were used to illustrate survival and survival rates were given as estimates. For LTx-free survival, only LTx was considered a competing risk. Every other analysis considered death or LTx as competing events. Patients with RAS-Inhibitors at baseline were considered as takers for the whole follow-up.

### Ethics

This study followed the declaration of Helsinki and was approved by the ethics committee of Hannover Medical School (Ethics approval number Nr.7935_BO_K_2018). All included patients have provided written informed consent for analysis of their data.

## Results

### Analysis I

Overall, 1181 patients with that underwent paracentesis were considered for this study of whom 667 patients were included. Intake of RAS-Inhibitors was documented in 41 patients, while this was not the case in the remaining 626 individuals. The leading reason for admission was hydropic decompensation (65%), followed by TIPS-evaluation (18%) and LTx evaluation (14%). Overall, all patients with RAS-Inhibitor intake were on stable doses and received their medication at least > 2 weeks before baseline. Here, 23 patients had documented ACE-Inhibitor intake and 19 patients were on Angiotensin II receptor inhibitors.

Mean time to paracentesis from admission was 2 days and a mean of 5,5Liters of ascites were removed (RAS-Inhibitors 2 days vs. No RAS-Inhibitors 2 days, P = 0.85 and RAS-Inhibitors 5,7Liters vs. 5,4Liters, P = 0.37). Patients reveiced a mean of 47 g Albumin after their first paracentesis (RAS-Inhibitors 45 g vs. No RAS-Inhibitors 47 g, P = 0.45).

Regarding baseline characteristics, there were significant differences between both groups. Patients in the RAS-Inhibitor group were older (RAS-Inhibitors: 62 years vs. No RAS-Inhibitors: 56 years, P = 0.001), had lower MELD and INR (RAS-Inhibitors: 16points vs. No RAS-Inhibitors: 19points, P = 0.02 and RAS-Inhibitors: 1.39 vs. No RAS-Inhibitors: 1.55, P = 0.001), while CHE values were higher (RAS-Inhibitors: 2.88 vs. No RAS-Inhibitors: 2.13, P = 0.006) and type II Diabetes Mellitus was more frequent in this group (RAS-Inhibitors: 46% vs. No RAS-Inhibitors: 23%, P = 0.002) (Table 1). Furthermore, SBP at baseline was numerically more frequent in patients with RAS-Inhibitor intake (RAS-Inhibitors: 32% vs. No RAS-inhibitors: 19%, P = 0.07).

**Table 1:** Baseline parameters in the unmatched setting.

Parameter ± SD	RAS-inhibitors (n = 41)	No RAS-inhibitors (n = 626)	P
Female, n (%)	13 (32)	226 (36)	0.62
Underlying liver disease			
Alcoholic cirrhosis, n (%)	14 (34)	284 (45)	0.20
Viral hepatitis, n (%)	9 (22)	105 (17)	0.69
Other, n (%)	18 (44)	221 (35)	0.31
Presence of varices, n (%)	32 (78)	476 (76)	0.85
History of variceal bleeding, n (%) (%)	3 (7)	85 (14)	0.34
Mean arterial pressure, mmHg	79 ± 13	75 ± 14	0.26
Infection at baseline, n (%)	18 (44)	237 (38)	0.51
UTI, n (%)	3 (7)	45 (7)	1.00
SBP, n (%)	13 (32)	121 (19)	0.07
Other, n (%)	2 (5)	79 (13)	0.21
Age, years	62 ± 12	56 ± 11	**0.001**
MELD, points (time of first paracentesis)	16 ± 7	19 ± 7	**0.02**
MELD, points (time of admission)	16 ± 3	19 ± 4	**0.001**
ACLF at baseline, n (%)	3 (7)	30 (5)	0.45
Creatinine, µmol/l	125 ± 71	144 ± 103	0.23
eGFR, ml/min/1.73 m^2^	61 ± 23	51 ± 29	0.06
Bilirubin, µmol/l	67 ± 111	78 ± 146	0.11
INR	1.39 ± 0.25	1.55 ± 0.44	**0.001**
Leukocyte count, tsd/µl	7.2 ± 3.6	9.0 ± 0.71	0.13
Cholinesterase, n (%)	2.88 ± 1.32	2.13 ± 1.08	**0.006**
Sodium, mmol/l	137 ± 5	135 ± 5	**0.02**
ALT, IU/ml	35 ± 19	48 ± 69	0.36
Albumin, g/l	27 ± 6	26 ± 6	0.51
Diabetes mellitus, n (%)	19 (46)	144 (23)	**0.002**
Platelet count, tsd/µl	166 ± 142	139 ± 97	0.11
History of SBP, n (%)	14 (34)	127 (20)	**0.03**
Diuretics, n (%)	31 (76)	446 (71)	0.60
Presence of refractory ascites, n (%)	4 (9)	67 (11)	0.85
HCC, n (%)	2 (5)	31 (5)	0.13
NSBB, n (%)	18 (44)	196 (31)	0.12

RAS-inhibitors were Ramipril (n = 16, median dose: 5 mg/day), Enalapril (n = 7, median dose: 10 mg/day) and Candesartan (n = 19, median dose: 8 mg/day).

Significant values are in bold.

### Endpoints

**Figure 1:**
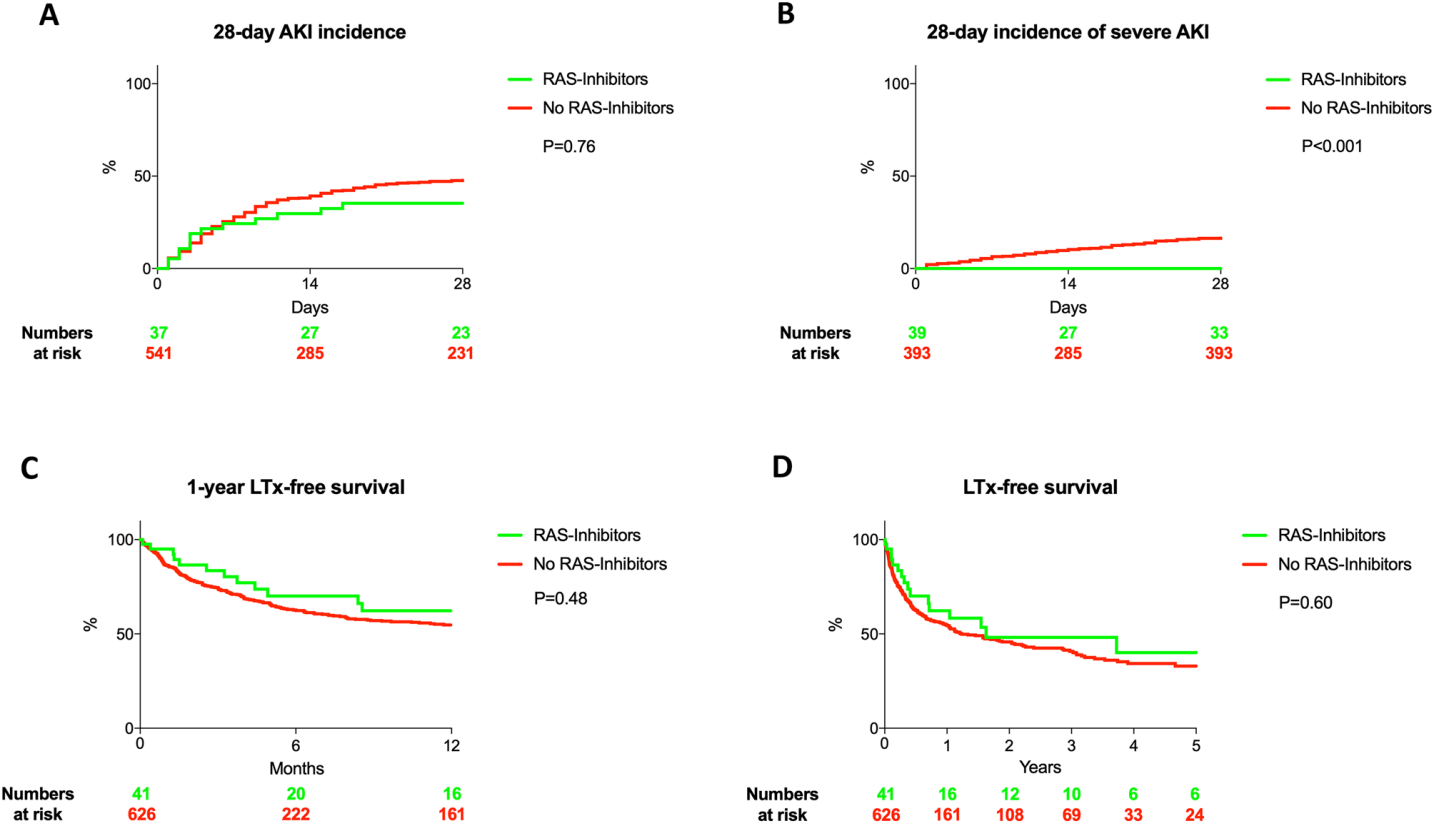
Unmatched cohort. 28-day incidences of AKI (**A**) and severe AKI (Grade III AKI, **B**). One year-LTx-free survival (**C**) and long-term survival (**D**).

Presence of AKI at baseline was comparable between both groups (RAS-Inhibitors: 12% vs. No RAS- Inhibitors: 12%, P = 0.94). Only MELD was independently associated with AKI within 28-days (HR: 1.06, 95%CI 1.04–1.09, P < 0.001) and intake of RAS-Inhibitors did not lead to a significant increased AKI rate (36% vs. 47%, HR: 0.92, 95%CI: 0.49–1.68, P = 0.76) (Table 2, Fig. 1A). In 4 of these patients RAS-Inhibitors were discontinued after AKI diagnosis. Two patients died before the AKI episode was resolved, in the other two patients, the RAS-Inhibitors were re-administered after discharge (mean time without RAS-inhibitors was 7 days). In terms of severe AKI, not a single event was observed in the RAS-Inhibitor group, hence multivariable analysis was not applied. However, log-rank analysis and Gray’s Test indicated a significantly lower rate of severe AKI in those with RAS-Inhibitors intake (0% vs. 17%, P < 0.001) (Fig. 1B). In a multivariable competing risk analysis of mortality, intake of RAS-Inhibitors was not associated, while age, MELD and leukocyte count were associated with the respective endpoint (28-day survival: 94% vs. 86%; 365-day survival: 62% vs. 55%, 5-year survival: 40% vs. 33%, RAS-Inhibitors: HR: 0.87, 95% CI: 0.50—1.49, P = 0.60, age: HR 1.02, 95% CI 1.01–1.03; P < 0.001, MELD: 1.04, 95% CI 1.02–1.06; P < 0.001 and HR: 1.03, 95%CI: 1.02–1.04, P < 0.001, respectively) (Table 2, Fig. 1C, D, Supplementary Fig. 2). RAS-Inhibitors were still not associated with mortality and AKI, even if eGFR and INR were used in the multivariable models instead of MELD (death: HR: 0.86, 95% CI: 0.48–1.55, P = 0.51; AKI: HR: 0.89, 95%CI: 0.44–1.77, P = 0.80).

**Table 2:** Results of the multivariable competing risk analyses.

Parameter	HR	95% CI	P
A: AKI—death/LTx are considered competing events			
Age, per year	1.02	1.01–1.03	** < 0.001**
MELD, per point	1.04	1.02–1.06	** < 0.001**
Platelet count, per 10^3^/µl	0.99	0.99–1.00	0.40
ACE/AT II, yes	0.87	0.50–1.49	0.60
Leucocyte count, per 10^3^/µl	1.03	1.02–1.04	< 0.001
Diabetes mellitus, yes	1.18	0.87–1.60	0.30
Mean arterial pressure, per mmHg	0.99	0.98–1.00	0.06
B: Survival—death is considered as a competing event			
Age, per year	1.00	0.99–1.01	0.88
MELD, per point	1.06	1.04–1.09	** < 0.001**
Platelet count, per 10^3^/µl	1.00	0.99–1.00	0.43
ACE/AT II, yes	0.91	0.49–1.68	0.76
Leucocyte count, per 10^3^/µl	1.02	0.99–1.05	0.14
Diabetes mellitus, yes	1.09	0.81–1.45	0.58
Mean arterial pressure, per mmHg	1.00	0.99–1.00	0.10

Significant values are in bold.

### Analysis II

For PPSM, patients without RAS-Inhibitors were matched with RAS-Inhibitors in a 3:1 manner. Matching factors included eGFR, age, leucocyte count, platelet count and presence of type II Diabetes Mellitus. After matching, all standardized mean differences were balanced (Supplementary Table 1) and baseline characteristics were comparable between both groups (Table 3).

**Table 3:** Baseline characteristics after propensity score matching.

Parameter ± SD	RAS-inhibitors (n = 39)	No RAS-inhibitors (n = 117)	P
Female, n (%)	12 (31)	37 (32)	0.92
Underlying liver disease			
Alcoholic cirrhosis, n (%)	14 (36)	47 (40)	0.67
Viral hepatitis, n (%)	8 (21)	13 (11)	0.20
Other, n (%)	17 (44)	57 (49)	0.61
Presence of varices, n (%)	31 (79)	98 (84)	0.55
History of variceal bleeding, n (%) (%)	3 (8)	17 (15)	0.29
Mean arterial pressure, mmHg	85 ± 16	81 ± 13	0.44
Infection at baseline, n (%)	15 (34)	36 (31)	0.37
UTI, n (%)	3 (8)	5 (4)	0.40
SBP, n (%)	11 (28)	20 (17)	0.14
Other, n (%)	2 (5)	11 (9)	0.43
Age, years	62 ± 11	62 ± 11	0.99
MELD, points (time of first paracentesis)	17 ± 7	17 ± 6	0.46
MELD, points (time of admission)	16 ± 1	16 ± 1	0.91
ACLF at baseline, n (%)	3 (8)	9 (8)	0.82
Creatinine, µmol/l	124 ± 71	134 ± 66	0.55
eGFR, ml/min/1.73m2	59 ± 15	59 ± 12	0.88
Bilirubin, µmol/l	45 ± 52	43 ± 64	0.25
INR	1.42 ± 0.25	1.40 ± 0.28	0.66
Leukocyte count, tsd/µl	7.3 ± 3.6	7.3 ± 5.6	0.95
Cholinesterase, n (%)	2.95 ± 1.28	2.48 ± 1.22	0.33
Sodium, mmol/l	137 ± 5	136 ± 5	0.47
ALT, IU/ml	34 ± 19	39 ± 43	0.25
Albumin, g/l	27 ± 6	28 ± 6	0.52
Diabetes mellitus, n (%)	18 (46)	52 (44)	0.83
Platelet count, tsd/µl	156 ± 130	157 ± 120	0.52
History of SBP, n (%)	12 (31)	25 (21)	0.24
Diuretics, n (%)	29 (74)	88 (75)	0.91
Presence of refractory ascites, n (%)	4 (10)	11 (9)	0.77
HCC, n (%)	2 (5)	7 (6)	0.85
NSBB, n (%)	18 (46)	39 (33)	0.36

### Endpoints

**Figure 2:**
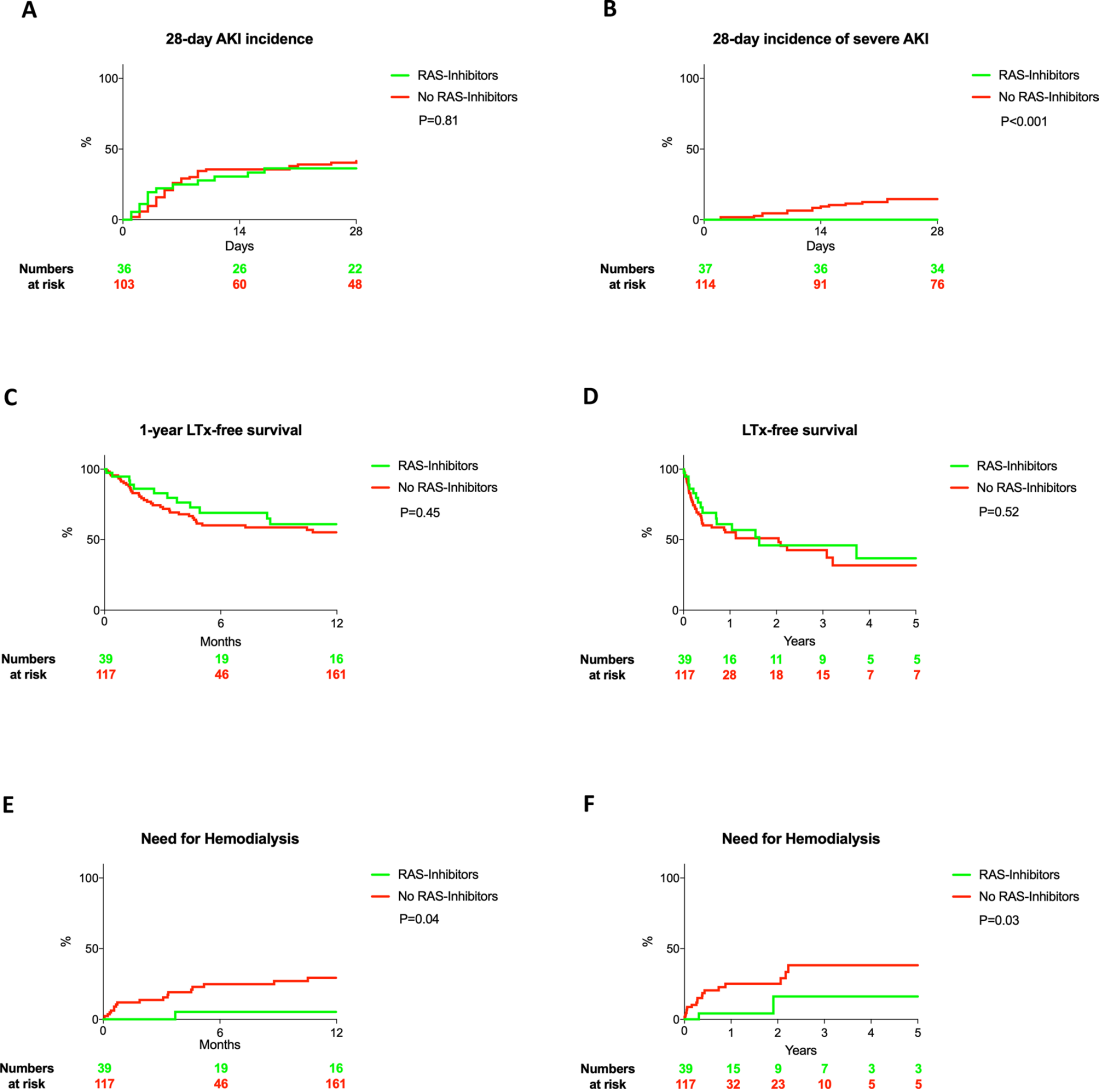
Matched cohort. 28-day incidence of AKI (**A**) and severe AKI (Grade III AKI, **B**). One year-LTx-free survival (**C**) and long-term survival (**D**). One year need for hemodialysis (**E**) and need for hemodialysis (**F**).

Overall, 28-day incidence of AKI did not differ between both groups (36% vs. 42%, HR: 0.92, 95%CI 0.49–1.74, P = 0.81). As mentioned above, no case of severe AKI was observed in the RAS-Inhibitor group and the incidence of severe AKI was significantly increased in those without RAS-Inhibitors intake (0% vs. 15%, P < 0.001) (Fig. 2A, B). In total, 16 AKI episodes were observed in those with RAS-Inhibitor intake and 12 patients still were on RAS-Inhibitors to the time of AKI diagnosis. 28-day, one year and long-term LTx-free survival were comparable between those with and without RAS-Inhibitor intake (28-day: 94% vs. 89%, P = 0.55, 1-year: 61% vs. 55% and 5-year: 37% vs. 32%; HR: 0.83, 95% CI: 0.48–1.45, P = 0.52, Fig. 2C,  D; Supplementary Fig. 3). Need for hemodialysis within one year and within 5 years was significantly decreased in patients with RAS-Inhibitor intake (1-year: 5% vs. 29% and Long-term: 16% vs. 38%; HR: 0.22, 95% CI 0.05–0.91, P = 0.03) (Fig. 2E, F). We further stratified patients according to their eGFR: in patients with eGFR > 60 (20 patients with RAS-Inhibitor intake vs. 57 without RAS-Inhibitor intake), RAS-Inhibitor intake was not associated with a decreased 28-day incidence of AKI, severe AKI or LTx-free survival (HR: 1.76, P = 0.46, P = 0.10, and HR: 0.66, P = 0.42, respectively). In those with eGFR < 60 (19 patients with RAS-Inhibitor intake vs. 60 without RAS-Inhibitor intake), RAS-Inhibitor intake was not associated with a decreased 28-day AKI incidence of lower 28-day LTx free survival (HR: 0.51, P = 0.18 and HR: 1.06, P = 0.86). However, the risk of developing severe AKI within 28-days was significantly decreased in patients with RAS-Inhibitor intake (P < 0.001) (data not shown).

**Figure 3:**
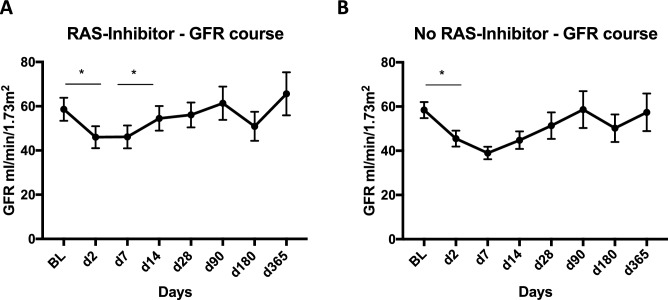
Matched cohort. Course of eGFR within the first year after baseline in patients with RAS-inhibitors (**A**) and without RAS-inhibitors (**B**).

The eGFR dropped significantly after baseline in the RAS-Inhibitors cohort, but recovered after 14 days. Within 365-days no further significant eGFR change was observed (eGFR course—BL: 59 ml/min/1.73 m^2^, d2: 46 ml/min/1.73 m^2^, d7: 46 ml/min/1.73 m^2^, d14: 55 ml/min/1.73 m^2^, d28: 56 ml/min/1.73 m^2^, d90: 61 ml/min/1.73 m^2^, d180: 51 ml/min/1.73 m^2^, d365: 66 ml/min/1.73 m^2^, Fig. 3). In patients without RAS-Inhibitor intake there also was a statistically significant eGFR drop documented within the first days. Afterwards, eGFR recovered in the observed collective after 28–90 days (eGFR course—BL: 59 ml/min/1.73 m^2^, d2: 46 ml/min/1.73 m^2^, d7: 39 ml/min/1.73 m^2^, d14: 45 ml/min/1.73 m^2^, d28: 51 ml/min/1.73 m^2^, d90: 59 ml/min/1.73 m^2^, d180: 50 ml/min/1.73 m^2^, d365: 57 ml/min/1.73 m^2^). Leucocyte values remained stable over the course of decompensation in patients with RAS-Inhibitors, while they increased between day 7 and day 14 in those without RAS-Inhibitors (d7: 7.57 tsd/µl vs. d14: 8.94 tsd/µl, P = 0.03, Supplementary Fig. 4).

Regarding de-novo HCC incidence, there was no significant difference in patients with RAS-Inhibitors intake compared to those without RAS-Inhibitors intake (HR: 0.81, 95% CI 0.17–3.72, P = 0.78, Supplementary Fig. 5). Moreover, 28-day incidence of infections did not differ significantly between those with RAS-Inhibitor intake and patients without RAS-Inhibitor intake (RAS-Inhibitor: 67% vs. No RAS-Inibitor intake: 57%, P = 0.28) (Supplementary Fig. 6). Of all documented infections, SBP was the most frequent infection (RAS-Inhibitors: 50% vs. No RAS-Inhibitors: 37%) followed by urinary tract infection (RAS-Inhibitors: 18% vs. No RAS-Inhibitors: 21%). Last, 28-day incidence of acute-on-chronic liver failure (ACLF) was not did not differ between patients with and without RAS-Inhibitor intake (RAS-Inhibitors: 24% vs. No RAS-Inhibitors: 24%, P = 0.29, Supplementary Fig. 7).

## Discussion

Decompensated liver cirrhosis is often accompanied by deterioration of kidney function. Development of AKI is associated with longer hospital stays and prognosis worsens with increasing severity of AKI^15,16^. Recent studies have elucidated the importance of comedication on renal endpoints in the setting of decompensated liver cirrhosis, however the role of RAS-Inhibitors remains a matter of debate^17^. In this study we investigated for the first time, how the intake RAS-Inhibitor impacts the short- and long-term incidence of clinical endpoints. To the time of hepatic decompensation, RAS-Inhibitor intake was not associated with an increased incidence of AKI or elevated mortality. Importantly, severe AKI and need for hemodialysis were significantly decreased in the respective group of patients.

The most common use of RAS-Inhibitors is the treatment of hypertension in the general population. In addition to the antihypertensive properties, other pleiotropic effects of RAS-Inhibitors have been suggested such as antifibrotic effects in patients with earlier stages of liver disease^18,19^. Of note, RAS-Inhibitors have previously been associated with improved survival in those with HCC or with a significant reduction of hepatic venous pressure gradient in CHILD A patients^8,20^. As of now, evidence was mostly limited to the setting of liver fibrosis or compensated cirrhosis. While we did not observe a decreased mortality in individuals with RAS-Inhibitors, other important endpoints were significantly decreased like the onset of severe AKI or long-term need for hemodialysis. Patients with decompensated liver cirrhosis and ascites are already progressed in their disease stage. Hence, positive effects on survival may only be present in those with earlier stages of cirrhosis, i.e. patients with CHILD A cirrhosis. Nonetheless, the positive association with renal endpoints were still detectable in the short-term and in the long-term follow-up.

One previous study from 1985 investigated the impact of newly administered Captopril in 6 patients with liver cirrhosis and ascites. The authors observed a reduction of mean arterial pressure and eGFR^10^. RAS-Inhibitors decrease Angiotensin II levels and ultimately prohibit the vasoconstriction in the vas efferens of the glomerulum. Thus, the intraglomerular hydrostatic pressure and overall blood pressure decrease, which is nephroprotective in those with hypertension or diabetes^21^. Since renal blood flow and arterial pressure is already decreased in the setting of decompensated liver disease and especially ascites^13^, current guidelines do not recommend general usage of RAS-Inhibitors in patients with ascites^11^. In contrast to the above-mentioned study, all patients in our cohort with RAS-Inhibitors were on a stable dose before decompensation and it was not newly administered. Importantly, no increased incidence of AKI was observed and episodes of severe AKI were significantly decreased in the short-term follow-up. Studies in the general population have shown that eGFR drops can be transient after initiation of RAS-Inhibitors^22^. Therefore, blood pressure of our patients could have already compensated RAS-Inhibitor intake as mean arterial pressure was comparably high in our cohort. Nonetheless, activation of the renin-angiotensin system is a physiological mechanism to counter hypotension and preserve renal blood flow^21^. Thus, deleterious effect of RAS-Inhibition cannot be ruled out in those with present AKI or hypotension, i.e. mean arterial pressure < 65 mmHg.

In this study, most patients had preserved kidney function, given mean eGFR was 59 ml/min/1.73 m^2^ in the matched setting. While those with and without RAS-Inhibitors experienced an eGFR drop after first paracentesis, eGFR recovered after 14 to 28 days. Of note, recovery seemed to be faster in those with RAS-Inhibitor intake. Importantly, medication was discontinued in approximately half of the patients who experienced AKI in the RAS-cohort. Discontinuation of RAS-Inhibitors leads to a rapid increase in blood pressure and could have therefore prevented further deterioration in those at risk of progressive AKI^23^. Since patients with decompensated liver disease have vulnerable hemodynamics, we do not recommend starting RAS-Inhibitor intake in the setting of hepatic decompensation as the risk of renal injury or hypotension is high^10^. However, while eGFR decreased in both, patients with and without RAS-Inhibitor intake after decompensation, long term effects of RAS-Inhibitor intake such as lower rates of hemodialysis have to be acknowledged. In this context, a decompensation per se should not be the trigger to discontinue RAS-inhibitor intake in those with cirrhosis.

Besides the hemodynamic effect of RAS-Inhibitors, immunomodulatory properties have been discussed^24,25^. Inflammation is generally present in patients with decompensated liver cirrhosis and plays a crucial role in the genesis of kidney injury^26^. In this setting, activation of Angiotensin type 1 receptors is involved in inflammation and induction of oxidative stress^27^. Use of ACE-Inhibitors and AT II receptor antagonists have been associated with increased levels of Angiotensin 1–7 which decreased inflammatory response^28^. Hence, RAS-Inhibitors may have mitigated the inflammatory response in the course of decompensation. This could explain, why patients with RAS-Inhibitors had a comparably stable leucocyte values, while those without RAS-Inhibitors showed increasing leucocyte values in the process. Moreover, administration of RAS-Inhibitors has already been associated with reduction the hepatovenous pressure gradient, one of the main drivers of decompensation^20,29,30^. Therefore, extrarenal effects of RAS-Inhibitors might also explain our findings, even if the lower incidence of renal endpoints did not translate into a higher LTx-free survival or a lower ACLF incidence in this cohort. Besides incidence of ACLF, incidence of HCC did also not differ. Potential antifibrotic properties of RAS-Inhibitors may not play such an important role in the setting of decompensated advanced chronic liver disease. On the other hand, our cohort of patients with RAS-Inhibitor intake was comparably small. Hence, group differences could have been missed.

Our study has some important limitations. First, the retrospective, non-randomized nature of this study could have led to some undetected group differences. Even if we adjusted for confounding factors via multivariable analysis or PPSM and important baseline factors like presence of refractory ascites, volume drained or presence of SBP were comparable, it cannot be ruled out that some undetected group differences remained even after matching. Furthermore, we only had a comparably small cohort of 41 patients with RAS-Inhibitor intake. This also limits the analysis in the eGFR stratified groups. Thus, some effects of RAS-Inhibitors may have been missed through underpowering. Furthermore, the effects of RAS-Inhibitors in patients with AKI could not be investigated, since patient numbers were to small. This is also the reason why ACE-Inhibitors and AT-2 Antagonists were not analyzed separately. To allow general statement, future studies need to validate the findings of our study. Moreover, data regarding the type of AKI was not available in many patients in this retrospective setting.

In conclusion, intake of RAS-Inhibitors resulted in a significantly decreased short-term incidence of severe AKI and long-term need for hemodialysis in patients with decompensated liver cirrhosis. Therefore, occurrence of ascites per se may not justify discontinuation of RAS-Inhibitors in the absence of renal failure or hypotension.

### Supplementary Information

Supplementary Information.

## Data Availability

TLT has access to all relevant data and vouches for the integrity of the work as a whole. The datasets used during the current study are available from the corresponding author on reasonable request.
