# Supplementary material for: Effects of renin–angiotensin inhibitors on renal function and the clinical course in patients with decompensated cirrhosis
Source: Sci Rep. 2023 Oct 14;13:17486. doi: 10.1038/s41598-023-44299-w (PMC10576780; doi:10.1038/s41598-023-44299-w)
Supplement: Supplementary file 1 — Supplementary Information. [file 41598_2023_44299_MOESM1_ESM.docx]

**- Supplementary Material –**

**Effects of Renin Angiotensin System inhibition on renal function and the clinical course in patients with decompensated cirrhosis**

*Tammo L. Tergast^1^, Marie Griemsmann^1^, Heiner Wedemeyer^1,2^, Markus Cornberg^1,2,3^, Benjamin Maasoumy^1,2^*

*1: Department of Gastroenterology, Hepatology and Endocrinology, Hannover Medical School, 30625 Hannover, Germany*

*2: German Centre for Infection Research, HepNet Study-House of the German Liver Foundation, 30625 Hannover, Germany*

*3: Centre for Individualised Infection Medicine (CiiM), 30625 Hannover, Germany*

**Table of content Page**

Supplementary Table 1 3

Supplementary Figure 1+2 4

Supplementary Figure 3+4 5

Supplementary Figure 5+6 6

Supplementary Figure 7 7

| **Parameter** | **SMD before matching** | **SMD after matching** |
| --- | --- | --- |
| Age | 0.55 | 0.01 |
| Presence of Diabetes Mellitus | 0.46 | 0.03 |
| GFR | -0.41 | -0.04 |
| Platelet count | 0.19 | -0.04 |
| Leukocyte count | -0.52 | 0.06 |
| Mean arterial pressure | 0.27 | -0.02 |

**Supplementary Table 1:** Standardized Mean Differences before and after matching.

**S1**

S1: Patient flow and application of in- and exclusion criteria.
